# The effects of Zinc on human body, including on renal failure and renal transplantation

**Published:** 2012

**Authors:** S Paun, M Tudosie, R Petris, R Macovei

**Affiliations:** * Clinic Emergency Hospital, Bucharest; ** “C.I. Parhon” National Endocrinology Institute

**Keywords:** Zn, toxicity, renal failure

## Abstract

Zinc (Zn) is an important element in human body and in the last period of time there were a lot of studies regarding its importance. It is significant for the good working of many organs. A special attention was given to the importance of the serum Zn in patients with renal failure. Among the micronutrients, zinc may rank with iron with regard to its importance for public health. This article reviews some epidemiological, clinical, diagnostic and therapeutic aspects of these conditions.

## Introduction

Zinc is essential for life, in particular for growth and development, through its role in hundreds of zinc enzymes and thousands of zinc proteins. The catalytic, structural, and regulatory functions in these proteins affect metabolism, gene expression, and signal transduction, including neurotransmission. Compared to several other metal ions with similar chemical properties, zinc is relatively harmless. Only exposure to high doses has toxic effects, making acute zinc intoxication a rare event. In addition to acute intoxication, long-term, high-dose zinc supplementation interferes with the uptake of copper. Hence, many of its toxic effects are in fact due to copper deficiency. Rather than being a toxic metal ion, zinc is an essential trace element. Whereas intoxication by excessive exposure is rare, zinc deficiency is widespread and has a detrimental impact on growth, neuronal development, and immunity, and in severe cases, its consequences are lethal. Zinc deficiency caused by malnutrition and foods with low bioavailability, aging, certain diseases, or deregulated homeostasis is a far more common risk to human health than intoxication.

## Zinc Homeostasis

The human body contains 2–3 g zinc, and nearly 90% is found in muscle and bone [**1**]. Other organs containing estimable concentrations of zinc include prostate, liver, the gastrointestinal tract, kidney, skin, lung, brain, heart, and pancreas [**2,3**]. Oral uptake of zinc leads to absorption throughout the small intestine and distribution subsequently occurs via the serum, where it predominately exists bound to several proteins such as albumin, α- macroglobulin, and transferrin [**4**]. On the cellular level, 30–40% of zinc is localized in the nucleus, 50% in the cytosol and the remaining part is associated with membranes [**5**]. Cellular zinc underlies an efficient homeostatic control that avoids accumulation of zinc in excess. Two protein families mediate the cellular homeostasis of zinc; the zinc-importer (Zip; Zrt-, Irt-like proteins) family, containing 14 proteins that transport zinc into the cytosol, and the zinc transporter (ZnT) family, comprising 10 proteins transporting zinc out of the cytosol [**6**]. Finally, metallothioneins (MTs) play a significant role in zinc homeostasis by completing up to 20% of the intracellular zinc [**7,8**] One MT molecule can bind up to seven zinc ions. Dynamic regulation of cellular zinc by MT results from the synthesis of the apo-form thionein (T) in response to elevated intracellular zinc levels by triggering the metal response element-binding transcription factor (MTF)-1 [**9**]. In addition, oxidation of cysteine residues can alter the number of metal binding thiols, connecting redox and zinc metabolism.

### Exposure to Zn

There are three major routes of entry for zinc into the human body; by inhalation, through the skin, or by ingestion [**10**]. Each exposure type affects specific parts of the body. Inhalation of zinc-containing smoke generally originates in industrial processes like galvanization, primarily affecting manufacture workers. In addition, military smoke bombs contain zinc oxide or zinc chloride, making soldiers a group in which several cases of inhalation of zinc-containing fumes were described. In these cases, soldiers developed adult respiratory distress syndrome (ARDS). Zinc chloride is generally caustic, so the effects could have risen from the specific properties of the compound, rather than being a direct effect of zinc intoxication. The most widely known effect of inhaling zinc-containing smoke is the so-called metal fume fever (MFF), which is mainly caused by inhalation of zinc oxide. This acute syndrome is an industrial disease which mostly occurs by inhalation of fresh metal fumes with a particle size <1 µm in occupational situations such as zinc smelting or welding [**12**]. Symptoms of this reversible syndrome include fever, muscle soreness, nausea, fatigue, and respiratory effects like chest pain, cough, and dyspnea [**11**]. The respiratory symptoms have been shown to be accompanied by an increase in bronchiolar leukocytes [**12**,**13**]. Dermal exposure: dermal exposure to zinc does not constitute a noteworthy toxicological risk. In contrast to a potentially harmful effect of zinc on skin (irritation), it should be noted that zinc is a well-known supplement for topical treatment of wounds and several dermatological conditions [**14****-****16**]. Oral exposure: Due to its nature as an essential trace element, oral uptake of small amounts of zinc is essential for survival. The recommended dietary allowance (RDA) for zinc is of 11 mg/day for men and 8 mg/day for women [**17**]. Lower zinc intake is recommended for infants (2–3 mg/day) and children (5–9 mg/day) because of their lower average body weights [**17**]. Immediate symptoms after an uptake of toxic amounts of zinc include abdominal pain, nausea, and vomiting. Additional effects include lethargy, anemia, and dizziness [**18**].

### Zinc-Induced Copper Deficiency

Taking up large doses of supplemental zinc over extended periods of time is frequently associated with copper deficiency [**19,20**]. This correlation seems to be caused by the competitive absorption relationship of zinc and copper. Dietary intake of different doses of copper and zinc did not significantly alter the absorption of the other metal, as long as they were given in the same ratio, irrespective of 1 mg/kg copper and 5 mg/kg zinc, or up to 36 mg/kg copper together with 180 mg/kg zinc were given [**21**]. Nevertheless, copper absorption is depressed when zinc is given in high excess over copper [**22**]. Frequent symptoms of copper deficiency include hypocupremia, impaired iron mobilization, anemia, leucopenia, neutropenia, decreased super oxide dismutase (SOD) (particularly erythrocyte SOD (ESOD)), ceruloplasmin as well as cytochrome-c oxidase, but increased plasma cholesterol and LDL: HDL cholesterol and abnormal cardiac function [**23,24**].

### Zinc Supplementation and Cancer

Zinc is not generally considered a causative agent for cancer development. In contrast, displacement of zinc from zinc-binding structures, i.e., finger structures in DNA repair enzymes, may even be a major mechanism for carcinogenicity of other metals such as cadmium, cobalt, nickel, and arsenic. One well investigated example in which an involvement of zinc in cancer development was suggested is prostate cancer. Notably, zinc levels in prostate adenocarcinoma are significantly lower than in the surrounding normal prostate tissues, suggesting an implication of zinc in the pathogenesis and progression of prostate malignancy. Men with moderate to higher zinc intake may have a lower risk for prostate cancer, but the opposite may be true at extremely high doses and long-term supplementation.

### Zinc Deficiency

Severe zinc deficiency can be either inherited or acquired. The most severe of the inherited forms is acrodermatitis enteropathica, a rare autosomal recessive metabolic disorder resulting from a mutation in the intestinal Zip4 transporter [**25**]. Symptoms of this condition include skin lesions, alopecia, diarrhea, neuropsychological disturbances, weight loss, reduced immune function, as well as hypogonadism in men, and can be lethal in the absence of treatment [**26**]. Acquired severe zinc deficiency has been observed in patients receiving total parental nutrition without supplementation of zinc, following excessive alcohol ingestion, severe malabsorption, and iatrogenic causes such as treatment with histidine or penicillamine [**27**]. The symptoms are mostly similar to those arising during acrodermatitis enteropathica. Clinical manifestations of moderate zinc deficiency are mainly found in patients with low dietary zinc intake, alcohol abuse, malabsorption, chronic renal disease, and chronic debilitation. Symptoms include growth retardation (in growing children and adolescents), hypogonadism in men, skin changes, poor appetite, mental lethargy, delayed wound healing, taste abnormalities, abnormal dark adaptation, and energy [**27**]. Moderate zinc deficiency can also occur as a consequence of sickle cell disease [**28**].

### Zn, renal failure and renal transplantation

There were studies which followed Zn metabolism in patients with renal transplantation and functioning allograft up to 96 months after the transplant. They concluded that subnormal plasma and hair zinc, as well as hyperzincuria, were present in patients less than 12 months post transplant. In contrast, patients who were more than 12 months post transplant had plasma zinc levels, hair zinc, and urinary zinc excretions in the normal range. Zinc concentrations in plasma and hair of some patients who were more than 12 months post transplant with renal failure, were subnormal and were similar to those in hemodialysis patients. These results suggest that abnormalities of zinc and taste persist up to 12 months post transplant and may be related to increased urinary zinc losses [**29**].

Zinc deficiency, abnormal taste acuity and decreased caloric intake may contribute to poor growth in children and adolescents with chronic progressive renal disease. Zinc supplementation on patients in chronic renal failure, but not yet on dialysis or in need of a transplant increased red blood cell zinc concentrations and taste acuity and in those with less advanced renal failure (serum creatinine less than 5.0 mg/dl) it also improved caloric intake. No changes in growth velocity were seen [**30**].

It is well known that sexual dysfunction is very common in patients with chronic kidney disease (CKD), but it is still significantly understudied. Studies have showed that phosphodiesterase-5 inhibitors (PDE5i) significantly increased the overall International Index of Erectile Function-5 (IIEF-5) score, oral zinc improved end of treatment testosterone levels and they are promising interventions for treating sexual dysfunction in men with CKD. However, plasma luteinizing and follicle-stimulating hormone levels were not changed at the end of the study period with zinc therapy and evidence supporting routine use of phosphodiesterase-5 inhibitors (PDE5i) and oral zinc in chronic kidney disease patients is limited [**31**]. Increased urinary zinc excretion is a cofactor for nephrogenic systemic fibrosis (NSF) / nephrogenic fibrosing dermopathy (NFD), a recently described disease, occurring only in patients with variable degrees of renal failure (RF) previously exposed to gadolinium-based contrast agents (GBCAs) for magnetic resonance imaging [**32**].

Plasma Zinc is lower in patients with chronic renal failure (CRF) but after the kidney transplantation, during the postoperative period, the zinc level returns to normal. Hemodialysis did not alter blood plasma zinc concentration [**33**]. Other studies reported basal serum Zn levels of patients with chronic renal failure (CRF) who were similar to those observed in normal. A decrement in serum Zn was recorded during Cyclosporine A infusion and on the first day after the surgery, followed by a slight and slow upward trend [**34**]. Plasma zinc concentrations were significantly decreased in patients under conservative renal treatment and hemodialysed patients [**35**].

## Conclusions

Zinc is an essential trace element, and the human body has efficient mechanisms, both on systemic and cellular levels, to maintain homeostasis over a broad exposure range. Consequently, zinc has a rather low toxicity, and a severe impact on human health by intoxication with zinc is a relatively rare event.

Whereas there are only anecdotal reports of severe zinc intoxication, zinc deficiency is a condition with broad occurrence and potentially profound impact. Here, the applications of “negative zinc”, *i.e.*, substances or conditions that deplete the body of zinc, constitute a major health risk. The impact ranges from mild zinc deficiency, which can aggravate infections by impairing the immune defense, up to severe cases, in which the symptoms are obvious and cause reduced life expectancy.
